# Recent progress in diagnosis and treatment of Human African Trypanosomiasis has made the elimination of this disease a realistic target by 2030

**DOI:** 10.3389/fmed.2022.1037094

**Published:** 2022-11-03

**Authors:** Andrés Álvarez-Rodríguez, Bo-Kyung Jin, Magdalena Radwanska, Stefan Magez

**Affiliations:** ^1^Laboratory for Biomedical Research, Ghent University Global Campus, Incheon, South Korea; ^2^Laboratory of Cellular and Molecular Immunology, Vrije Universiteit Brussel, Brussels, Belgium; ^3^Department of Biomedical Molecular Biology, Ghent University, Ghent, Belgium; ^4^Department of Biochemistry and Microbiology, Ghent University, Ghent, Belgium

**Keywords:** trypanosomiasis, treatment, LAMP, gambiense HAT, rhodesiense HAT, CRISPR-Cas, diagnostics

## Abstract

Human African Trypanosomiasis (HAT) is caused by unicellular flagellated protozoan parasites of the genus *Trypanosoma brucei*. The subspecies *T. b. gambiense* is mainly responsible for mostly chronic anthroponotic infections in West- and Central Africa, accounting for roughly 95% of all HAT cases. *Trypanosoma b. rhodesiense* results in more acute zoonotic infections in East-Africa. Because HAT has a two-stage pathogenesis, treatment depends on clinical assessment of patients and the determination whether or not parasites have crossed the blood brain barrier. Today, ultimate confirmation of parasitemia is still done by microscopy analysis. However, the introduction of diagnostic lateral flow devices has been a major contributor to the recent dramatic drop in *T. b. gambiense* HAT. Other techniques such as loop mediated isothermal amplification (LAMP) and recombinant polymerase amplification (RPA)-based tests have been published but are still not widely used in the field. Most recently, CRISPR-Cas technology has been proposed to improve the intrinsic diagnostic characteristics of molecular approaches. This will become crucial in the near future, as preventing the resurgence of HAT will be a priority and will require tools with extreme high positive and negative predicted values, as well as excellent sensitivity and specificity. As for treatment, pentamidine and suramin have historically been the drugs of choice for the treatment of blood-stage gambiense-HAT and rhodesiense-HAT, respectively. For treatment of second-stage infections, drugs that pass the blood brain barrier are needed, and melarsoprol has been effectively used for both forms of HAT in the past. However, due to the high occurrence of post-treatment encephalopathy, the drug is not recommended for use in *T. b. gambiense* HAT. Here, a combination therapy of eflornithine and nifurtimox (NECT) has been the choice of treatment since 2009. As this treatment requires IV perfusion of eflornithine, efforts were launched in 2003 by the drugs for neglected disease initiative (DNDi) to find an oral-only therapy solution, suitable for rural sub-Saharan Africa treatment conditions. In 2019 this resulted in the introduction of fexinidazole, with a treatment regimen suitable for both the blood-stage and non-severe second-stage *T. b. gambiense* infections. Experimental treatment of *T. b. rhodesiense* HAT has now been initiated as well.

## Introduction

Human African Trypanosomiasis (HAT) and Animal African Trypanosomosis (AAT) are two diseases that affect the development of potentially highly productive agricultural areas in sub-Saharan Africa. Both diseases result from exposure to a range of pathogenic salivarian trypanosomes, of which the first recorded infection was reported in India, not Africa. In 1880 Dr. Griffith Evans reported the discovery of *Trypanosoma evansi*, in horses and camels suffering from “Surra” disease (1). Not long after that, tsetse flies were identified as the main transmitter of *Trypanosoma brucei* in Africa, discovered by Sir David Bruce as the causative agent of the disease called ‘Nagana’ (2). After that, trypanosomes were confirmed to be responsible for “sleeping sickness” in humans. The discovery of *T. brucei rhodesiense* and *T. brucei gambiense* as the causative agents of HAT in East- and West-Africa, respectively followed soon after (3–5). Subsequently, other pathogenic insect-transmitted animal trypanosomes were described, including *T. congolense* and *T. vivax*, and a sexually transmitted parasite, i.e., *T. equiperdum*, was found to specifically affect equines. Interestingly, salivarian trypanosomes are unique parasites in the sense that despite their unicellular small size, they live freely in the blood and lymphatics of their host, without using any intracellular “hiding” mechanisms that would protect them from being attacked by the host’s immune system (6). They are able to do so, as they have developed an intricate system of antigenic variation of their surface coat that allows trypanosomes to evade elimination by antibodies and other adaptive immune system components. Indeed, the trypanosome surface is made up of a densely packed protein coat consisting of variant surface glycoprotein (VSG) molecules that provides not only a physical barrier against antibody/complement attacks, but also allows for rapid clearance of immune molecules bound to the surface (7, 8). The efficiency of this system is the reason why today there is not a single field applicable vaccine available that can provide significant protection against HAT or AAT. A second mechanism that contributes to the success of trypanosomes as extracellular parasites, is their capacity to inflict serious damage to the host B cell compartment. Indeed, trypanosomes have developed a mechanism by which they can ablate vaccine-induced memory responses, independent of the vaccine target’s specificity (9–12). This means that while antigenic variation offers short-term protection against the adaptive immune system by constantly evading infection-induced antibodies, the destruction of immunological memory provides trypanosomes with long-term protection against the host immune system. Interestingly, while chronic trypanosome infections do cause significant pathology and will eventually lead to the death of the host if left untreated, the parasite does have a self-regulating growth control mechanism that prevents excessive parasitemia. This quorum sensing is mediated by the recognition of di- and tri-peptides that result from proteolytic activity of parasite-secreted enzymes (and possibly host proteases as well) (13). Hence, when a threshold level of protein degradation is reached, dividing parasites, so called long slender bloodstream form in case of *T. brucei*, undergo a phase of growth arrest, and differentiate into stumpy form parasites that metabolically prepare for uptake by the tsetse vector. Through this mechanism, peak parasitemia levels do not overwhelm the host, and transmission can occur under the most favorable conditions. While the biological mechanisms driving quorum sensing in trypanosomes are currently not fully unraveled, the practical consequence of the well-controlled low parasitemia levels is that development of field applicable point-of-care diagnostics is not easy. Hence, as will be outlined below, current diagnostic tests mostly rely on the detection of host antibodies that cross-react with parasite antigens, as detection of the parasite itself is much more difficult. This in itself leads to a situation where theoretic parameters such as sensitivity and specificity often blur the reality of the applicability of a test under field conditions. Low disease prevalence only exacerbates this problem. To fully grasp the issue at hand, the following two equations should be taken into account:

$$S\,e\,n\,s\,i\,t\,i\,v\,i\,t\,y=\frac{\Sigma \,T\,r\,u\,e\,\left(+\right)}{\Sigma \,T\,r\,u\,e\,\left(+\right)+\Sigma \,f\,l\,a\,s\,e\,\left(-\right)}$$

$$S\,p\,e\,c\,i\,f\,i\,c\,i\,t\,y=\frac{\Sigma \,T\,r\,u\,e\,\left(-\right)}{\Sigma \,T\,r\,u\,e\,\left(-\right)+\Sigma \,f\,l\,a\,s\,e\,\left(+\right)}$$

It is obvious that under conditions of low prevalence and low numbers of true positive cases, the sensitivity of an assay only reflect the absence of false negatives, without giving any information about the correctness of a positive test score. Similarly, low prevalence conditions will automatically result in high true negative results. Hence, the high specificity value of a given test only reflects the relatively low number of false positive results, without giving any information about the validity of a positive test outcome. Hence in terms of reliability in finding truly infected HAT patients, neither of these values are very useful. Indeed, only the positive predictive value (PPV) and negative predictive value (NPV) are useful parameters that allow to take an active intervention decision based on an individual diagnostic test result.

$$\text{PPV}=\frac{\text{Σ}\,\text{True}\,\left(+\right)}{\text{Σ}\,\text{True}\,\left(+\right)+\text{Σ}\,\text{flase}\,\left(+\right)}$$

$$\text{NPV}=\frac{\text{Σ}\,\text{True}\,\left(-\right)}{\text{Σ}\,\text{True}\,\left(-\right)+\text{Σ}\,\text{flase}\,\left(-\right)}$$

**FIGURE 1:**
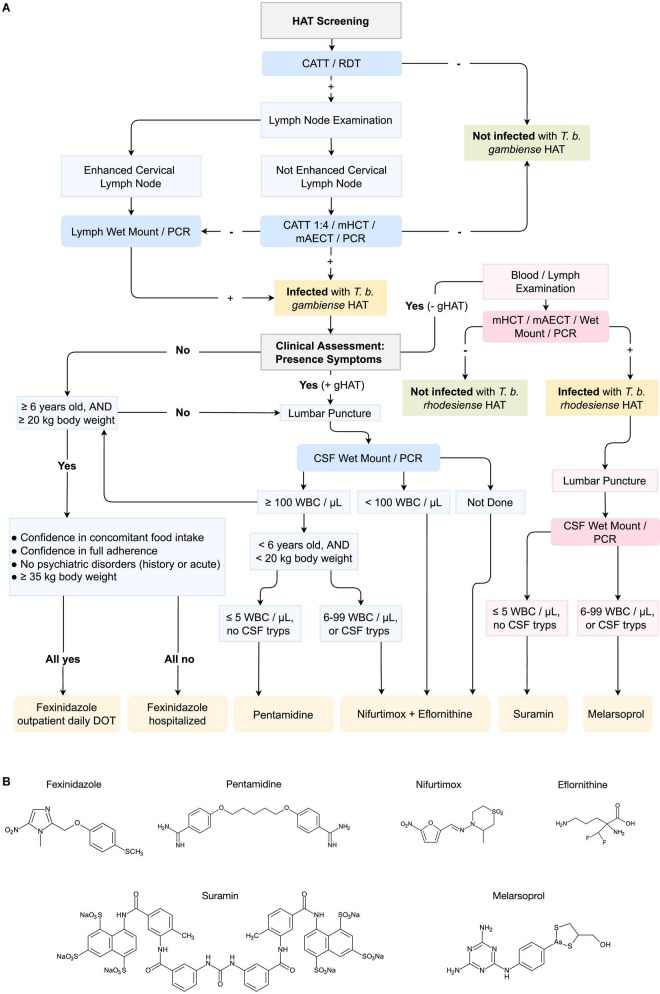
Pipeline for the diagnosis and treatment of *T. b. gambiense* and *T. b. rhodesiense* Human African Trypanosomiasis (HAT) infections. **(A)** The pipeline includes the diagnosis of HAT through CATT (Card Agglutination Test), RDT (Rapid Diagnosis Test), mHCT (microhaematocrit centrifugation), mAECT (mini-anion exchange centrifugation), PCR (Polymerase Chain Reaction) or other amplification techniques, and CSF (Cerebrospinal Fluid) Wet Mount. DOT (Directly Observed Treatment); WBC (White Blood Cell). In blue, pipeline for the diagnosis of *Trypanosoma brucei gambiense* HAT, and in pink for *T. b. rhodesiense* HAT. Recommended drugs for treatment are displayed in orange. Sequence adapted from Geneva: World Health Organization, WHO interim guidelines for the treatment of gambiense Human African Trypanosomiasis, 2019 (101); and Tiberti N., Hainard A. and Sanchez J. C., Translation of Human African Trypanosomiasis biomarkers toward field application, 2013 (105). **(B)** Chemical formulation of the principal drugs used for the treatment of HAT (molecular structure artwork was kindly provide by Prof. Dr. ir. Philippe Heynderickx of the Ghent University Global Campus).

Today, antibody-based screening tests excel in their sensitivity and specificity. They do however suffer from a low PPV, meaning that with every positive test, a confirmation is required before a drug treatment decision is taken (14). Making the treatment decision of HAT in particular even more complex, is the fact that as outlined above, human infective trypanosomes can cross the blood brain barrier. As the latter imposes specific limitations with respect to drugs that have been available until recently, HAT diagnosis requires both parasite detection and disease staging. However, with the recent introduction of fexinidazole for HAT treatment, this staging requirement can be largely overcome. An overview of the complete diagnosis and treatment decision pipeline is provided at the end of this review in Figure 1.

## The card agglutination test for Human Animal Trypanosomiasis and the adaptation to an Ab-detecting lateral flow immunochromatographic assay

Human African Trypanosomiasis diagnosis may differ considerably depending on the *T. brucei* subspecies. The high parasite numbers observed in *T. b. rhodesiense* symptomatic infections allow for simply basing its diagnosis on clinical signs along with microscopy (15, 16). The most widely used and simplest microscopy methods, such as the examination of wet blood smear or capillary tube centrifugation, generally allow for analytical sensitivities ranging from 500 to 10,000 trypanosomes/ml (15) (Table 1). In contrast, *T. b. gambiense* infections may result in parasite loads of 1–10 trypanosomes/ml, thus requiring a method with improved analytical sensitivity (16). Since its development in 1978, the Card Agglutination Test for Trypanosomiasis (CATT) has become the standard approach for the initial screening and diagnosis of *T. b. gambiense* HAT in the field (16, 17). The CATT serodiagnostic test is based on the detection of anti-parasite antibodies in the blood, plasma or serum, both in a transient or in an established infection (18, 19). While several methods with the same principle have been developed using different *T. b. gambiense* antigens (Ag), the most widely used test contains the lyophilized *T. b. gambiense* LiTat 1.3 and LiTat 1.5VSGs (17, 20). Despite its widespread use and huge contribution toward the control of HAT, the CATT test has numerous drawbacks. This has led to the search for new and improved serological methods (i.e., antibody or antigen based tests). Actually, CATT is used in both active and passive screening, but is not truly optimized for use in the field (i.e., rural health facilities) (21, 22). First, it lacks robustness, as (i) it is mainly manufactured at a single research institute (Institute of Tropical Medicine, Antwerp, Belgium), limiting the total available supplies, and (ii) it requires cold storage in order to avoid spoiling (16, 21). Secondly, CATT is not equipment free, as it needs electricity (capillary centrifugation) for its optimal execution, resulting in some operational challenges (23). Third, the test is packed in a multiple dose format (CATT-R250 of 50 doses or CATT-D10 of 10 doses) with a useful life of a single day, limiting its use as a screening test for individual patients (21). Finally, the CATT sensitivity and specificity values range between 87 and 98% (15) (Table 1), but can be affected by (i) a low parasitemia, or (ii) the setting, as patients from different places can be infected with different strains of *T. b gambiense*, some lacking or not expressing the LiTat 1.3 gene (15, 24, 25). Considering the current situation of HAT prevalence (less than 0.1%), the occurrence of a relatively large number of false negative cases will not impact the overall negative predictive value (NPV) of the test, reaching 99% (6, 26). However, the opposite situation arises for the positive predictive value (PPV) of the test, when large number of false positive cases occur (26). Indeed, even with a CATT sensitivity of 98% and specificity ranging from 98 to 99.9%, the field observed PPV can range from an extremely poor 5% to a mere 50%. In true “field terminology” that means that a positive CATT score provides only a 5–50% chance that a patient is truly positive. Hence, given the toxicity of HAT treatments, a positive CATT result must always be confirmed by microscopy detection of trypanosomes in body fluids (blood, lymph node aspirate, or cerebrospinal fluid), sometimes requiring an mini ion exchange chromatography step to enrich the parasites in the analytical sample (21, 27). In view of the aforementioned, it is clear that CATT does not meet the ASSURED criteria, nor the recently implemented REASSURED criteria for point-of-care (POC) diagnostic testing (28).

**TABLE 1:** Intrinsic properties of the main different methods for the diagnosis of Human African Trypanosomiasis (HAT).

	Limit of detection	Sensitivity (%)	Specificity (%)
**Microscopy-based methods**			
Examination of wet blood	10,000 parasites/mL	5–55	100
Blood film (Giemsa staining)	–	25–50	100
Examination lymph node aspirate	–	20–60	100
Capillary tube centrifugation (or Woo)	500 parasites/mL	45–90	100
Mini-anion-exchange centrifugation technique	50 parasites/mL	75–90	100
**Serology-based methods**			
Card agglutination test for trypanosomiasis (CATT)	–	87–98	98
HAT Sero-K-SeT (Coris BioConcept, Gembloux, Belgium)	–	98–100	97–98
SD BIOLINE HAT (Standard Diagnostics, Kyonggi-do, South Korea)	–	89	95
SD BIOLINE HAT 2.0 (Standard Diagnostics, Kyonggi-do, South Korea)	–	70–88.2	98
Ag-based LFIA*	–	80	92
**Molecular-based methods**			
Polymerase chain reaction (PCR)	1–1,000 parasites/mL	87–100	92–98
Loop-mediated isothermal amplification (LAMP)	1–1,000 parasites/mL	75–90	95–100
Recombinase polymerase amplification (RPA)**	100 parasites/mL	93	100
PCR + CRISPR/Cas***	10–1,000 parasites/mL	82–100	100
LAMP + CRISPR/Cas****	5,000 virus/mL	83	100
RPA + CRISPR/Cas*****	360–2,500 parasites/mL	73–94	94–100

Adapted from Bottieau and Clerinx (15).

*Data from infections of Animal African Trypanosomiasis (AAT) (32). Tests with this technique are not yet available for HAT.

**Data from infections of AAT (64) and Chagas Disease (63). Tests with this technique are not yet available for HAT.

***Data from infections of Tegumentary Leishmaniasis (103). Tests with this technique are not yet available for HAT.

****Data from infections of COVID-19 (104). Tests with this technique are not yet available for HAT.

*****Data from infections of Malaria (80, 81). Tests with this technique are not yet available for HAT.

In order to improve this situation, several rapid diagnostic tests (RDTs) for HAT have been developed and introduced in the field in the recent years (22, 23, 26, 27, 29). Most notable are the lateral flow immunochromatographic assays (LFIAs), which are based on the detection of target analytes (antibodies in this case) contained in a liquid sample (i.e., body fluids) (30). These targets are displaced through capillary forces along a nitrocellulose strip, where molecules that detect the presence of the analytes are attached (30). Commercialized LFIAs for HAT are Sero-K-SeT (Coris BioConcept, Gembloux, Belgium), SD BIOLINE HAT 1.0 and SD BIOLINE HAT 2.0 (Standard Diagnostics, now Abbott Diagnostics Inc., Gyeonggi-do, South Korea), which show promising sensitivities (98–100, 89, and 70–88.2%, respectively) and specificities (97–98, 95, and 98%, respectively) (Table 1) (15). However, as the first two above-mentioned LFIAs use native LiTat1.3 and LiTat1.5 VSGs for antibody detection, this has resulted in scale-up problems (23, 31). To solve this issue, the SD BIOLINE HAT 2.0 uses recombinantly produced LiTat1.3 and LiTat1.5 VSG variants (23). Unfortunately, as the immunological principle of the VSG-based LFIAs and CATT are the same, the problem outlined above with respect to the low PPV is not solved by the introduction of this new technology. Thus, the use of LFIAs for antibody detection also does not solve the requirement for active parasite detection as confirmation for every positive test score. Therefore, despite sensitivity and specificity being similar to those of CATT, and its improved applicability in the field (fully complying with the REASSURED criteria for POC diagnosis), LFIAs also have several drawbacks (26, 27). Probably the most dramatic issue at hand is the fact that as is the case for all Ab-based diagnostics, HAT-LFIAs are inefficient when it comes to differentiating between current or past infections, or to performing post-treatment follow-ups (18). In contrast, Ag-based tests do address this limitation, offering a drastic improvement in the diagnosis of HAT. These, compared to Ab-based tests, often show much better PPV, but generally have lower sensitivity (depending on the amount of antigens in the blood) (32). Still, new detection technologies such as the use of Nanobodies, can overcome this problem (32). Despite its great potential, there is currently no Ag-test available for HAT.

## Polymerase chain reaction, loop mediated isothermal amplification, and recombinant polymerase amplification as new molecular solutions in diagnosis

**FIGURE 2:**
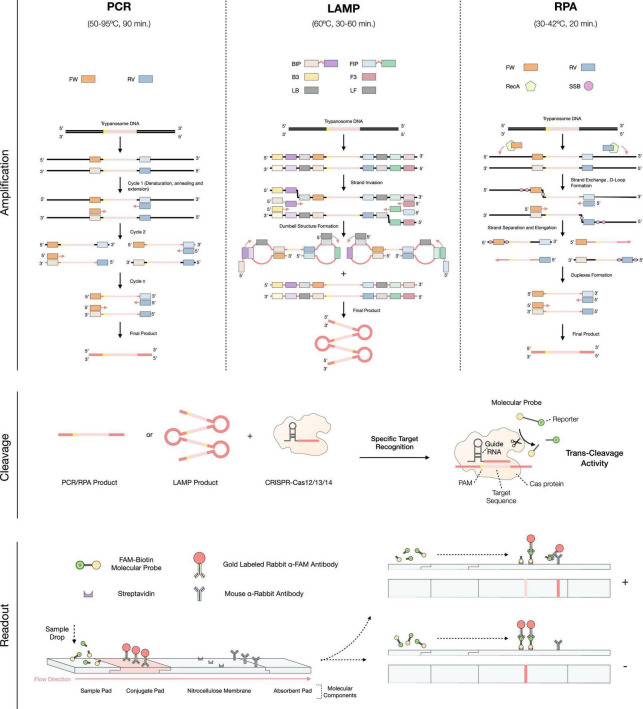
Overview of the implementation of the CRISPR-Cas technology to the molecular-based diagnosis for Human African Trypanosomiasis (HAT) infections. The first step consists in the amplification of the target DNA/RNA through PCR, LAMP, or RPA. In PCR: FW (Forward Primer) and RV (Reverse primer); In LAMP: BIP (Backward Inner Primer), FIP (Forward Inner Primer), B3 (Backward 3 Primer), F3 (Forward 3 Primer), LB (Loop Backward Primer) and LF (Loop Forward Primer); in RPA: FW (Forward Primer), RV (Reverse Primer), RecA (Recombinase A), and SSB (Single Stranded Binding Protein). Squares matching the same colors as the primers but with a lighter shade correspond to the complementary sequence of these. Then, the cleavage process occurs, by applying the CRISPR-Cas technology to the amplification products. Finally, the readout procedure, which is based on the detection of the molecular probes, can be performed by lateral flow immunoassay.

Despite recent advances in diagnostic and treatment techniques for *T. b. gambiense* infections, confirmation of positive cases by detection of the parasite in body fluids is still necessary. This is of vital importance especially in passive screening situations, where in the case of failure to re-confirm infection, a false positive result would force the patient to be exposed to potentially toxic medication (16). While today microscopy observations are still most commonly used for test result validation, DNA or RNA amplification methods have been standardized in laboratories with more resources (33, 34). One of these methods is polymerase chain reaction (PCR), a widely used approach for the molecular detection of HAT. An overview of its amplification principle can be found in Figure 2. The main Trypanozoon-specific (*T. brucei*, *T. evansi*, and *T. equiperdum*) PCR targets are the satellite DNA called the *Trypanosoma brucei* repeat (TBR) (35), the 18S ribosomal RNA gene (36) and the spliced leader (SL) sequence of the parasite’s mRNA (37). The currently used subspecies-specific markers are the serum resistance-associated (SRA) gene for *T. b. rhodesiense* (38) and the *T. b. gambiense-*specific glycoprotein gene (TgsGP) for *T. b. gambiense* (39). Unlike Trypanozoon-specific targets, the latter are single-copy genes, which restricts the limit of detection (LOD) and sensitivity of the test, leading to a higher number of false negatives (40). Even so, PCR is sensitive and specific, with values of up to 100 and 98%, respectively, and a LOD of 1–1,000 parasites/mL (Table 1), directly comparable with the Mini anion exchange centrifugation/microscopy technique (15). However, PCR still does not possess the status of POC test, due to its high cost, complexity, reaction time (around 1–2 h), necessity of controlling the reaction temperatures (by using a thermal cycler), requirement of skilled personnel, etc. (41, 42). Therefore, this molecular based method is only suitable for laboratory-based testing (i.e., passive screening) and not POC testing (i.e., active screening) (43).

Loop-mediated isothermal amplification (LAMP) is a more recently developed technique, capable of solving the main problems of PCR, and allowing the use of a molecular test in POC (44, 45). Its main characteristic is the isothermal nature of the reaction (i.e., single constant temperature) that only requires a simple, low-cost heat source (e.g., a portable heat block or a water bath) (46). An overview of its amplification principle can be found in Figure 2. The best validated DNA Trypanozoon-specific target for LAMP is the repetitive insertion mobile element (RIME), which used to be commercially available as Loopamp *T. brucei* Detection Kit (Eiken Chemical, Tokyo, Japan) (47, 48). Subspecies-specific LAMP tests for the detection of both *T. b. gambiense* and *T. b. rhodesiense* infections have also been developed, using the TgsGP and SRA genes as targets, respectively (49, 50). Other isothermal amplification methods exist [e.g., NASBA (51), HDA (52), Smart-AMP (53), etc.], however, most have certain drawbacks that limit them for use as POC tests. Some of these problems include (i) undesirable non-specific amplifications (false positives, lower specificity), (ii) the need for cumbersome template purification methods, (iii) suboptimal temperature and time reaction conditions, and (iv) the susceptibility of the amplification enzymes to inhibitory factors in clinical specimens, leading to difficulties in performing a correct and quantitative readout (41, 46, 48, 54). LAMP is a highly sensitive and specific technique (with mean values of 90 and 95%, respectively), similar to PCR (55). However, its reaction time is faster, generally requiring 60 min. to have the highest amplification products, while 30 min. is sufficient to have a visible signal when a decreased LOD is acceptable (56, 57). Reaction rates can be further improved by optimizing concentrations of dNTPs and Mg^2+^ (54). Also, the addition of loop primers dramatically reduces the time needed for LAMP reactions to reach the optimal fluorescence threshold (from 33 to 8 mins), thus also reducing the overall reaction time (54). In general, LAMP does not lead to non-specific amplifications (54), and allows the detection of 10–1,000 parasites/mL in blood. If genetic material is extracted and purified, loop primers are added, and multi-copy gene targets are used, this can be improved down to 1 parasites/mL (Table 1) (49, 54, 58). Hence, the LAMP LOD matches the numbers for PCR, but offers the advantage of being performed isothermally. Indeed, this test can be performed at a constant temperature of 58–65°C. Furthermore, LAMP allows the amplification to be performed using clinical specimens without resulting in inhibition of their enzymes (41, 57). In addition, it permits a direct readout either by fully quantitative (and more subjective) techniques such as checking turbidity due to magnesium pyrophosphate precipitate in the reaction tube, or by more qualitative and user-friendly methods such as LFIAs (54, 59, 60). Despite these features, LAMP also has some drawbacks. For example, and in the same way as PCR, since they are very sensitive techniques, the slightest contamination of a negative sample with a positive sample will probably result in a false-positive result (41). With regards to the reaction time, despite being acceptable, processing time is still not optimal for the efficient parallel diagnosis of a large number of samples.

Recombinase polymerase amplification (RPA) is an even more recently developed isothermal technique. An overview of its amplification principle can be found in Figure 2. RPA allows DNA amplification to be performed at even lower temperatures than PCR and LAMP, namely at a constant temperature of 30–42°C (61). This temperature resembles the body temperature of a normal, healthy adult human being, so incubation of the test could be directly performed holding the test in the hand, underarm or other adequately warm body area (62). Reaction time also improves over other molecular-based tests, being generally 20 min (61). While there is not yet an RPA test for HAT, RPA tests have been developed for AAT and Chagas disease, with sensitivities of 93%, specificities of 100% and LOD of 100 parasites/mL (Table 1) (63, 64). Similarly to LAMP, RPA can be directly performed on serum samples, in the presence of potential biochemical inhibitors such as hemoglobin, ethanol, or heparin (65). Moreover, its readout can be performed in a LFIA format, improving POC use (64). However, unlike LAMP, RPA often produces non-specific amplification even in water controls, an effect that has been reported by the original inventors of the technology (54). Finally, it should be noted that RPA kits are currently commercialized by a single company, TwistDx [now part of Abbott Laboratories, US (66)] which has full control over the price and availability of the kits. RPA has not yet been approved by the food and drug administration (FDA), and for now is intended for research-only applications (65, 67).

## CRISPR-Cas innovation and its potential in Human African Trypanosomiasis diagnostics

Since the emergence of CRISPR-Cas technology 10 years ago, its applications in the field of parasitology, including HAT, have been substantial (68–71). For instance, the technology has enabled the development of several programmed genome-editing approaches for *T. brucei* parasites (71). The technology is based on the use of the RNA-guided DNA endonuclease Cas, which can be programmed to target a specific DNA/RNA sequence by using a synthetic guide RNA (gRNA) fragment. Additionally, Cas is able to cut both target DNA strands (RNA-complementary and non-complementary), resulting in double-strand breaks (DSBs) (68). While most CRISPR-Cas applications use the Cas9 endonuclease as a working tool, other members of the same family, with more advanced applications, have recently been discovered (72–74). Specifically, some members of this CRISPR-Cas family (Cas12, Cas13, and Cas14) have apart from their inherent endonuclease activity, additional *non-specific*, *collateral* or *trans*-cleavage activity (75). As a result of the DNA/RNA target recognition and cut, the activated Cas protein undertakes a *non-specific* cleavage of surrounding off-target single stranded DNA/RNAs (76, 77). This property has been successfully used as a sensitive diagnostic method to specifically detect nucleic acids present in a sample. The reaction requires an initial step of amplification of the DNA/RNA target, either by isothermal or traditional PCR methodologies, as well as the addition ssDNA/RNA molecular probes containing a reporter (to be cleaved by Cas), allowing for the detection of the target (Figure 2) (75, 77). Depending on the Cas protein and amplification method, this technique is referred to as: (1) Specific high-sensitivity enzymatic reporter unlocking (SHERLOCK), by using Cas13a and RPA pre-amplification (73); (2) DNA endonuclease-targeted CRISPR trans reporter (DETECTR), by using Cas12a and RPA pre-amplification (73); (3) a one-hour low-cost multipurpose highly efficient system (HOLMES), by using Cas12a and PCR pre-amplification (78); and (4) a one-Hour Low-cost Multipurpose highly Efficient System Version 2 (HOLMESv2), by using Cas12b and LAMP pre-amplification (79). Through these different techniques, it has been possible to develop highly sensitive and specific CRISPR-based diagnostic tests for several diseases, including malaria (80, 81), Ebola (82), SARS-CoV-2 (83), Zika and Dengue (84). When used for parasitic disease detection, CRISPR-Cas based molecular diagnosis allows to improve the sensitivity of the test up to 100-fold compared to traditional PCR and delivers specificity values of up to 100% (Table 1). In terms of diagnostic applicability at the POC level, CRISPR-Cas technology is perfectly suited as a molecular-based test when coupled to a LFIAs readout (Figure 2), with an estimated final cost of $0.61 (USD) per test (80, 85). Moreover, given the current situation of HAT prevalence (where it is crucial to improve the PPV of diagnostic tests) this technology offers a possible solution, by greatly reducing the number of false positive results due to non-specific amplification (54). However, these new diagnostic tests have also some shortcomings that need to be addressed in the future, such as the nucleic acid extraction and, only in some cases, their purification. The latter forces the test to be performed in multiple steps, prolonging reaction times. In turn, this leads to a higher risk of contamination and worsens the user-friendliness of the procedure, thus not fulfilling all REASSURED criteria (28, 86). Nonetheless, several strategies that allow the test execution in a single one-pot reaction mixture, including target pre-amplification coupled with CRISPR-Cas target detection, have already been published (87, 88). In conclusion, given the promising results obtained so far with CRISPR-Cas based technologies, it seems reasonable to expect the development of such methods for the molecular diagnosis of HAT and AAT in the near future.

## Conclusion with respect to Human African Trypanosomiasis diagnosis

The combination of rapid antibody screening and microscopy based diagnosis has had a tremendous impact of HAT control over the last decade. The current approach to HAT surveillance will remain in place in the foreseeable future, but as the incidence of human infections will further diminish, there will be a need for new diagnostic tools. These should excel by an improved PPV as compared to the current LFIA tests. Hence, it is suggested to develop new affordable molecular tools that can be implemented as POC tests, and would be able to correctly diagnose patients in a one-step procedure without interference by their past trypanosome exposure history. Isothermal amplification technology (i.e., RPA or LAMP), possibly enhanced by combining it with a highly specific CRISPR-Cas step, would be able to fulfil all the requirements of a modern target product profile for HAT diagnosis. Such test should be capable of detecting both *T. b. gambiense* and *T. b. rhodesiense* HAT using a relatively short reaction time, so that patients could be correctly diagnosed while waiting for a treatment decision.

## Advances in the treatment of Human African Trypanosomiasis

Chemotherapy targeting trypanosomiasis goes back more than a hundred years, and in fact was at the cradle of the entire concept of chemotherapy as a solution for disease cure. Indeed, both trypan red and trypan blue were described in the early years of the 20th century as chemicals that could target trypanosomes, hence their names (89). While the former was found to exhibit a toxicity level that was too high to be considered as a useful drug, the latter showed a lack of efficacy in killing trypanosomes. However, this approach quickly gave rise to the discovery of a chemical homolog suramin, which has been in use to treat the blood-stage of trypanosomiasis at least since 1916 (90). The drug is still in use today for the intravenous (IV) treatment of *T. b. rhodesiense* HAT, but is limited in application as it is unable to cross the blood brain barrier. Hence, when *T. b. rhodesiense* infections enter the meningo-encephalitic stage, treatment options are restricted to the use of melarsoprol (91). This arsenic-containing compound is also to be administered through the IV route and unfortunately has a very narrow therapeutic index. In addition, its high toxicity requires that administration is done slowly, and distributed over at least a 10-day period of time. The high risk of developing post-treatment encephalitic reactions or encephalitic syndrome (ES) ranges from 5 to 18%, with up to half of these patients dying as a consequence of this complication (92). Today, *T. b. rhodesiense* infections in humans remain rare, but the% in the total HAT pool is increasing due to the tremendous success in controlling *T. b. gambiense* HAT that has been achieved over the last decade (93). Since the 1940s, gambiense-HAT has been successfully treated with pentamidine, a drug that can be administered by either intra-muscular or IV injection (94). As this compound fails to cross the blood brain barrier, second stage gambiense-HAT was initially treated the same way as rhodesiense-HAT, with melarsoprol, but the discovery of eflornithine offered a much safer alternative since the 1990s, however, it still requires IV infusion (95). Based on the success of eflornithine, and in the search of better and easier treatment regimens, eflornithine was later combined with nifurtimox, a drug that already had shown good results in the treatment of Chagas disease. This combinational therapy, referred to as NECT, in which oral nifurtimox treatment is combined with IV eflornithine administration, has been a major contributor to the success of the near-elimination of gambiense-HAT we witness today (96). Implementation of NECT was in large made possible by the efforts of the not-for-profit organization DNDi (Drugs for Neglected Diseases initiative), the organization that was also the driving force behind the introduction of the new break-through medication for gambiense-HAT, i.e., fexinidazole (97). This latest compound no longer requires invasive interventions as it can be in an oral-only approach. It is effective for the treatment of both the blood-stage and non-severe second-stage gambiense-HAT, making invasive procedures of stage determination unnecessary in most cases (98, 99). An update on trypanocidal pharmaceuticals and their resistances was recently published by Kasozi et al. (100), while all current treatment protocols and guidelines for HAT interventions are available through the WHO (101). To date, fexinidazole treatment is not yet approved for rhodesiense-HAT, but preparations for this application are underway (102). To improve user-friendliness, fexinidazole is supplied as a treatment regimen containing either fourteen 600 mg tablets for children, or 24 tablets for adults. Tablets must be taken with food, during or immediately after the main meal of the day, preferentially at the same time of day. Treatment starts with a loading dose over the first 4 days, followed by a maintenance dose over the next 6 days, resulting in a full treatment course of 10 days. Adverse reactions to fexinidazole include vomiting, nausea, asthenia, decrease appetite, headache, insomnia tremor and dizziness, with vomiting being more frequently in children than adults. Neuropsychiatric adverse reactions are more frequently observed in comparison to NECT treatment, hence the suggestion to keep patients with psychiatric disorders hospitalized during the 10-day treatment period.

## Conclusion with respect to Human African Trypanosomiasis treatment

The recent introduction of fexinidazole as an oral treatment for *T. b. gambiense* HAT has been a game-changer in the fight against sleeping sickness. Next, a similar success story is needed for the treatment of *T. b. rhodesiense* HAT. Finally, as the latest HAT treatments still require a 10-day treatment period, future efforts should focus on finding therapies that could result in successful cure in a shorter time, ultimately leading to the discovery of a 1-day/one-pill solution for both forms of HAT.

## Final conclusion

To date, there are still only two treatment options available for rhodesiense-HAT patients, including the treatments with suramin for early-stage infections and the very toxic arsenic-based melarsoprol treatment regimen for the meningo-encephalitic stage of the disease. Hence, while *T. b. rhodesiense* is not the major cause of human trypanosomiasis, further research is needed to improve treatment possibilities. In contrast, three tailored options are available for gambiense-HAT patients, and the recent introduction of fexinidazole is a major step forward in making disease elimination by 2030 a realistic target. Still, the diagnostic procedure preceding the treatment choice is cumbersome as shown in Figure 1. Indeed, as this procedure starts with a low-PPV test screening, parasitological confirmation is an absolute requirement. In case more severe clinical symptoms are present, a very invasive lumbar puncture is performed to assess the presence of parasites in the CSF, or detect an increase in white blood cell counts. Either of these can confirm that parasites have crossed the blood brain barrier, demanding NECT treatment. In other cases, fexinidazole treatment can be provided either to outpatient, or as a hospitalized care option, depending on the specific needs of the HAT victim. As outlined above, HAT diagnostics itself have made the transition over the last years from a possibly ambiguous agglutination test, to a more user-friendly LFIA. Still, improvements are needed in order to obtain POC HAT diagnostic devices that comply with the (RE)ASSURED criteria. The future implementation of isothermal amplification-based methods, and CRISPR-Cas methods could be the solution here. This is particularly important now that HAT prevalence rates have come down and less than 1,000 cases/year that are being reported to the WHO. This means that a diagnostic test format with a poor PPV, is becoming less-and-less useful in the field. In addition, taking into account the drawback of antibody testing procedures, it is clear that future test development has to focus on detection of the presence of the pathogen, through antigen detection or DNA identification. Indeed, the aim of HAT diagnostics over the next few decades is going to be to try and prevent the re-emergence of Human Trypanosomiasis, so that the success in fighting HAT over the last decade will not have been in vain.

## Author contributions

SM and AÁ-R drafted the manuscript. MR and B-KJ reviewed and amended the manuscript. AÁ-R provided most of the artwork. All authors contributed to the article and approved the submitted version.
